# Supplementary material for: Study on mechanical properties and permeability of elliptical porous scaffold based on the SLM manufactured medical Ti6Al4V
Source: PLoS One. 2021 Mar 4;16(3):e0247764. doi: 10.1371/journal.pone.0247764 (PMC7932120; doi:10.1371/journal.pone.0247764)
Supplement: Attachment — Submitted filename: Response to Reviewers.docx [file pone.0247764.s002.docx]

Dear Editors and Reviewers:

Thank you for the reviewers’ comments concerning our revision entitled “**Study on Mechanical Properties and Permeability of Elliptical Porous Scaffold Based on the SLM Manufactured Medical** **Ti6Al4V**” (ID: **PONE-D-20-36743R1**). Those comments are all valuable and very helpful for revising and improving our paper, as well as the important guiding significance to our researches. We have studied the comments carefully and have made correction which we hope meet with approval. Revised portion are marked in blue in the paper. The main corrections in the paper and the responds to the reviewer’s comments are as flowing:

Responds to the reviewer’s comments:

**Reviewer #1:**  (No Response)

**Reviewer #2:** Thanks for the author's effort in addressing the comments. Most of the comments have been properly addressed, but still have 2 minor ones left:

I would suggest to accept for publication after the 2nd revision is properly made.

(1)	In the response to original comment (7), you gave the maximum mesh element size. So, this means that the element size is not uniform for the model geometry, right? If so, please could you specify which factors / parameters control the actual element size? This would be useful for the other researchers if they want to replicate the model.

Response: Yes, the element size is not uniform for the model geometry. Because the geometry is not the same at every position, some parts are weaker, and some edge parts are finely divided. We defined the approximate global seed size as 0.03mm and the maximum size as 0.05mm. We understand that the meshing you mentioned will have an impact on the results. Since we conducted a mechanical compression test, the simulation results are more of an approximate reference. Of course, we have made amendments as required. (Page 6, Lines 119-121)

(2)	In the response to original comment (9), although it was claimed that revision was made, it seems like no changes in the manuscript (e.g. the 2nd term of NS equation is still incorrect; units of parameters in NS equation are wrong). I know this will not influence the final results, as you use the commercial CFD software. However, once it is written in the paper, you have to make sure it is correct. So, please be careful when do the revision.

Response: Thanks for your comments, we have revised it according to your comments. (Page 10, Lines 171-173)

We tried our best to improve the manuscript and made some changes in the manuscript. We appreciate for Editors/Reviewers’ warm work earnestly, and hope that the correction will meet with approval. Once again, thank you very much for your comments and suggestions.

Thank you and best regards.

Yours sincerely,

Chenglong Shi

Email: qhislcas@126.com
