# Supplementary material for: Study on mechanical properties and permeability of elliptical porous scaffold based on the SLM manufactured medical Ti6Al4V
Source: PLoS One. 2021 Mar 4;16(3):e0247764. doi: 10.1371/journal.pone.0247764 (PMC7932120; doi:10.1371/journal.pone.0247764)
Supplement: Attachment — Submitted filename: Response to Reviewers.docx [file pone.0247764.s003.docx]

Dear Editors and Reviewers:

Thank you for the reviewers’ comments concerning our revision entitled “**Study on Mechanical Properties and Permeability of Elliptical Porous Scaffold Based on the SLM Manufactured Medical** **Ti6Al4V**” (ID: **PONE-D-20-36743R2**). Those comments are all valuable and very helpful for revising and improving our paper, as well as the important guiding significance to our researches. We have studied the comments carefully and have made correction which we hope meet with approval. Revised portion are marked in blue in the paper. The main corrections in the paper and the responds to the reviewer’s comments are as flowing:

Responds to the reviewer’s comments:

**Reviewer #1:** (No Response)

**Reviewer #2:** Thanks for your time & effort on addressing my comments. My previous comment 1 has been addressed and changes were made in manuscript. That's good. However, the comment 2 was not addressed thoroughly. You did not revise eq. 4, which I have already mentioned twice in previous comments. According to that stated in paper, you want to express the incompressible flow. From continuity equation, the incompressible flow has the divergence of velocity of 0 (∇ * u= 0). However, what you wrote is an incomplete equation, advection of something unknown = 0. I don't know which scalar / vector you want to put in. But please use divergence of velocity = 0 (i.e. ∇ * u= 0) for incompressible flow in Eq. 4, if that is what you want to express. I know this minor problem will not influence the results, but it is better to be corrected before publishing.

Response: Thanks for your comments, we have studied the comments carefully and have made correction which we hope meet with approval. (Page 10, Lines 171-172)

We tried our best to improve the manuscript and made some changes in the manuscript. We appreciate for Editors/Reviewers’ warm work earnestly, and hope that the correction will meet with approval. Once again, thank you very much for your comments and suggestions.

Thank you and best regards.

Yours sincerely,

Chenglong Shi

Email: qhislcas@126.com
